# The effect of multimodal comprehensive care methodology training on oral health care professionals’ empathy for patients with dementia

**DOI:** 10.1186/s12909-021-02760-2

**Published:** 2021-06-03

**Authors:** Masaki Kobayashi, Mio Ito, Yasuyuki Iwasa, Yoshiko Motohashi, Ayako Edahiro, Maki Shirobe, Hirohiko Hirano, Yves Gineste, Miwako Honda

**Affiliations:** 1 Department of Geriatric Medicine, National Hospital Organization Tokyo Medical Center, Meguro-ku, Tokyo, 152-8902 Japan; 2 Gunma University Graduate School of Health Science, Maebashi-City, Gunma 371-8514 Japan; 3 Department of Dentistry, Haradoi Hospital, Fukuoka-City, Fukuoka 813-8588 Japan; 4 Tokyo Metropolitan Geriatric Hospital and Institute of Gerontology, Itabashi-ku, Tokyo, 173-0015 Japan; 5 IGM-France, 66250 Saint-Laurent-de-la-Salanque, France

**Keywords:** Empathy, Multimodal comprehensive care methodology, Dental care, Jefferson scale of physician empathy, Oral health assessment tool

## Abstract

**Background:**

The prevalence of oral diseases in people with dementia has increased, and patients with dementia have worse oral health than people without dementia. However, in the provision of oral care, these patients often exhibit care-resistant behaviours. Empathy is important for health care professionals who provide dental care for people with dementia. A study was conducted to assess whether a multimodal comprehensive care methodology training programme, Humanitude™, was associated with an improvement in empathy for people with dementia among oral health care professionals.

**Methods:**

This research was a pre-post prospective study. A total of 45 dentists and dental hygienists participated in a 7-h multimodal comprehensive care methodology training programme. Participants’ empathy for their patients was evaluated with the Jefferson Scale of Physician Empathy-Health Professionals Version (JSPE-HP) before the training and 1 month after the training (primary outcome). Each participant listed 3 patients with poor oral health due to the refusal of usual oral care or dental treatment from his or her clinical practice. The oral health of the 3 care-resistant patients listed by each participant was evaluated by the Oral Health Assessment Tool (OHAT) before the training and 1 month after the training (secondary outcome).

**Results:**

The post-training response rate was 87% (21 dentists and 18 dental hygienists). From pre-training to post-training, the multimodal comprehensive care methodology training significantly increased the mean empathy score (from 113.97 to 122.95, *P* < 0.05, effect size = 0.9). Regardless of gender, profession and years of clinical experience, all post-training subgroup scores were higher than the pre-training subgroup scores. The tongue, natural teeth, and oral hygiene scores of patients with dementia who resisted usual oral care or dental treatment, as assessed by the OHAT, were significantly improved compared with those before the training.

**Conclusions:**

The multimodal comprehensive care methodology training was associated with an improvement in oral health professionals’ empathy for patients with dementia. These findings suggest that randomized controlled trials with large sample sizes will be needed.

**Trial registration:**

UMIN Clinical Trials Registry (UMIN-CTR), UMIN000041687. Registered 4 September 2020 – Retrospectively registered, https://upload.umin.ac.jp/cgi-open-bin/ctr_e/ctr_view.cgi?recptno=R000047586

## Background

The prevalence of oral diseases in people with dementia has increased, and patients with dementia have worse oral health than people without dementia [1, 2]. Poor oral health can lead to pain, difficulty in food intake, impaired general health and reduced quality of life.

However, patients with dementia often exhibit care-resistant behaviour, as some studies have shown [3, 4]. Care-resistant behaviour refers to actions taken by an older adult to avoid receiving any type of assistance or care activity [5]. Oral health care-resistant behaviour is a fear-evoked response to a caregiver’s unintentionally threatening behaviour during oral care. A previous study described the neurobiological principles of threat perception and fear response during oral care in patients with dementia [6]. Another reported the effectiveness of strategies based on the neurobiology of threat perception in preventing and reducing care-resistant behaviours during oral care [7].

Empathy for patients with dementia has been suggested as one of the critical abilities required for health professionals [8]. In the health care setting, empathy can be defined as the ability to understand a patient’s experiences and feelings, as well as the ability to communicate this understanding [9, 10]. Therefore, it is important for oral health professionals to understand the concept of care-resistant behaviour and to increase their empathy for people with dementia. However, there is no practical training on caring for patients with dementia for oral health care professionals in Japan.

A French care methodology, namely, a multimodal comprehensive communication technique called Humanitude™, has been widely implemented in hospitals and nursing homes in European countries for 40 years [11]. This methodology is based on a humane philosophy that emphasizes respect for each person’s dignity, freedom and autonomy and that is based on the neurological principles of dementia, including the conception of care-resistant behaviour, as shown by a previous study [5]. The education programme focuses on skills for 4 elements of communication with patients: gaze, talk, touch, and assistance with standing up. Additionally, according to the methodology, all care is provided in a sequence consisting of 5 structures: 1) notification, 2) preparation, 3) integration of communication, 4) emotional consolidation, and 5) the next appointment [12].

Past studies have reported web-based empathy interventions in caregivers of people with dementia [13, 14] and dental students [15]. However, no previous study has been conducted on the development of empathy for patients with dementia among oral health care professionals.

We hypothesized that a multimodal comprehensive care methodology training programme would promote a positive change in the relationships between oral health care professionals and patients with dementia who resisted oral care or dental treatment through increased empathy as measured using the Jefferson Scale of Physician Empathy-Health Professionals Version (JSPE-HP) [16]. The aim of this study was to evaluate whether a multimodal comprehensive care methodology training programme would be associated with an improvement in oral health care professionals’ empathy for patients with dementia. We also examined a secondary outcome, i.e., oral health in patients with dementia, using the Oral Health Assessment Tool (OHAT) [17].

## Methods

### Study design, setting, and participants

A pre-post prospective study was conducted. This study adhered to the CONSORT guidelines. Dentists and dental hygienists who had a current active dental practice with people with dementia as patients were recruited through a website for research collaboration in September 2018. The on-site training was held in Fukuoka on November 23, 2018.

### Procedure

**Fig. 1:**
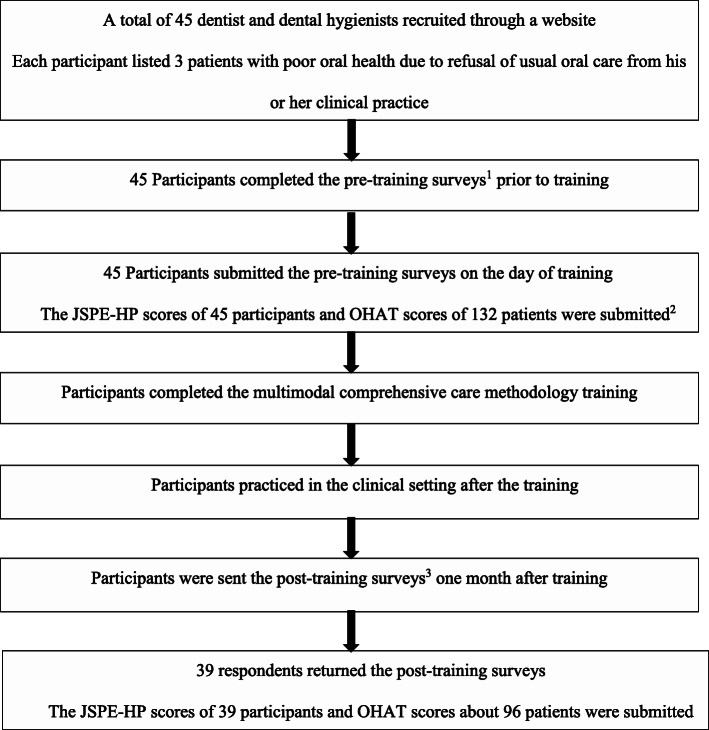
Procedure of multimodal comprehensive care methodology training. ^1^Pre-training surveys: completion of the Jefferson Scale of Physician Empathy-Health Professionals Version (JSPE-HP) and Oral Health Assessment Tool (OHAT) scores for 3 patients with poor oral health due to refusal of usual oral care or dental treatment prior to training. ^2^One participant could not submit information about patients. ^3^Post-training surveys: completion of the Jefferson Scale of Physician Empathy-Health Professionals Version (JSPE-HP) and Oral Health Assessment Tool (OHAT) scores for 3 patients one month after training

Each participant listed 3 patients with poor oral health due to the refusal of usual oral care or dental treatment from his or her clinical practice. Prior to the training, the participants completed the JSPE-HP and determined the OHAT scores for the patients with poor oral health due to the refusal of usual oral care or dental treatment. A 7-h multimodal comprehensive care methodology training programme was provided. One month after the training, secondary surveys were conducted to obtain the post-intervention JSPE-HP scores and post-intervention OHAT scores of the 3 patients whom each participant listed (Fig. 1).

### Instruments

#### Jefferson scale of physician empathy-health professionals version (JSPE-HP)

The JSPE was specifically developed to measure empathy in medical students (Student Version), health professionals (Health Professionals Version) and health profession students (Health Professions Student Version). In this study, the JSPE-HP was chosen. The questionnaire is a 20-item instrument that has been widely used and validated among health professionals and trainees. It uses a 7-point Likert scale anchored by “strongly disagree” and “strongly agree” (range: 20–140) [9, 18]. The JSPE has been translated into 25 languages. In this study, the Japanese version of the JSPE, which has been linguistically validated [19], was used. The participants completed the JSPE-HP. The scale was administered before the training (pre-training) and 1 month later (post-training).

#### Oral health assessment tool (OHAT)

The oral health of the identified patients who resisted usual oral care or dental treatment was assessed using the OHAT, an instrument developed specifically for use in residential care facilities [17]. The OHAT was chosen because the tool is simple to use and quick to administer based on visual inspection. The OHAT was also specifically developed for use in elderly patients with varying degrees of cognitive impairment. The tool is used to visually evaluate 8 items: the appearance of the lips, tongue, gums and tissues, and saliva; overall oral hygiene; the state of the natural teeth; the condition of dentures; and the presence of pain. The oral care methods and dental treatment differ depending on each OHAT item. The OHAT is composed of 8 items, each of which is assigned a score ranging from 0 (healthy) to 2 (unhealthy). The oral cavities of the patients were assessed using the OHAT prior to the study and 1 month after the intervention. The oral health assessor of each patient was the participant who identified him or her.

### Outcome measures

The primary outcome was the difference in the JSPE-HP scores from before the training (pre-training) to 1 month later (post-training). The secondary outcome was the difference in the OHAT scores between pre-training and post-training.

#### Multimodal comprehensive care methodology training programme

As an intervention, we implemented a French multimodal comprehensive care methodology training programme. In 1979, Gineste and Marescotti developed a multimodal comprehensive care methodology called the Humanitude™ care methodology [11, 12]. Humanitude™ is the set of particularities that allow us to feel that we are members of the human species and to recognize other human beings as members of the same species. Gineste and Marescotti developed this care methodology based on the considerations of dignity, freedom and autonomy in the daily care provided to dependent and vulnerable persons. The methodology focuses on 4 elements of communication with patients: gaze, talk, touch, and assistance with standing up. Additionally, all care is provided in a sequence consisting of 5 structures: 1) notification 2) preparation 3) integration of communication 4) emotional consolidation and 5) the next appointment. The aim of notification is to announce the presence of the caregiver, avoid surprise approaches and respect the patient’s privacy and autonomy. Preparation represents the initial establishment of a relationship through the relationship pillars (gaze, speech and touch), and it allows the caregiver to obtain consent for the relationship from the person receiving the care. Integration of communication includes the provision of care with a consistent positive emotional environment between the caregiver and the patient. Emotional consolidation is a stage of cognitive and mental stimulation that leaves a positive impression of the relationship and the care in the emotional memory of the person receiving it, facilitating consent to the relationship and acceptance of future care. The next appointment is the final moment of the relationship, in which commitment to future care is affirmed. At this stage, goodbyes are said, and a new meeting is scheduled, which prevents a feeling of abandonment [11]. The programme administered in the current study consisted of training participants in skills that can be used in dental care using the multimodal comprehensive care methodology. The training was performed by a certified instructor. The instructor provided lectures, demonstrations and role-play workshops to teach the participants how to adapt the methodology to patients who refused dental care.

### Statistical analysis

The normal distribution of all data was verified by the Shapiro-Wilk test. A paired-samples t-test was used to test for significant differences between the pre-training and post-training JSPE-HP scores. The baseline characteristics of the people with dementia who refused oral care or dental treatment were compared using Fisher’s exact test for categorical variables and the Mann-Whitney U test for continuous variables. The Wilcoxon signed rank test was used to test for significant differences in the categories of the OHAT scores, assessed as post-training data. The magnitudes of changes in the JSPE-HP score variables were computed as standardized mean differences (Cohen’s d measure of effect size), which express changes in standard deviation units. Values of 0.2, 0.5, and 0.8 have been suggested as indicating small, medium, and large effect sizes, respectively [20]. The internal consistency reliability was estimated using Cronbach’s alpha. These analyses were performed using R statistical software (version 3.5.3). Statistical significance was defined as a *P*-value < 0.05.

## Results

A total of 45 participants, including 26 dentists and 19 dental hygienists, were enrolled in the study to complete the multimodal comprehensive care methodology training programme. Prior to the training, the information of the 132 patients who refused oral care or dental treatment listed by the participants was obtained.

### Comparison of Jefferson scale of physician empathy (JSPE) scores

For the 45 participants, the post-training response rate was 87% (27 females and 12 males, 21 dentists and 18 dental hygienists). According to Gough and Hall, a response rate of at least 75% should be achieved to ensure the representativeness of the sample for surveys mailed to professionals [21].

The distribution of the participants and the results of the pre-training and the post-training JSPE scores are shown in Table 1. The JSPE-HP scores showed a statistically significant improvement (from 113.97 to 122.95, *P* < 0.05) from pre-training to post-training. The standardized effect size for these mean differences was large (effect size 0.9). Higher scores were observed regardless of gender, profession, and years of clinical experience. In particular, there were significant improvements in empathy among females, dentists, dental hygienists, participants with 11–20 years of experience, and participants with 21–30 years of experience (*P* < 0.05). Based on the Cronbach’s alpha, the internal consistency reliability was 0.61.

**Table 1:** Pre-training and post-training results of the Jefferson Scale of Physician Empathy

		Mean Score (95% CI)		*P-*value ^a^	Standardized Mean Difference in Change
		Pre-intervention	Post-intervention		
Jefferson Scale of Physician Empathy: Total Empathy	*N* = 39	113.97 (103.84 to 124.1)	122.95 (119.77 to 126.12)	***P*** **< 0.001**	0.90
Male	*N* = 12	115.50 (103.05 to 127.95)	121.75 (111.37 to 132.13)	0.07	0.55
Female	*N* = 27	113.30 (104.19 to 122.41)	123.48 (113.8 to 133.16)	***P*** **< 0.001**	1.10
Dentist	*N* = 21	114.76 (102.55 to 126.97)	122.86 (112.32 to 133.4)	**0.009**	0.73
Dental hygienist	*N* = 18	113.06 (105.81 to 120.31)	123.06 (113.9 to 132.22)	**0.001**	1.25
Less than 11 years of experience	*N* = 4	102 (96.9 to 107.1)	125 (113.62 to 136.38)	0.07	2.61
11–20 years of experience	*N* = 15	114.67 (103.21 to 126.13)	125.07 (114.11 to 136.03)	**0.002**	0.96
21–30 years of experience	*N* = 15	115.47 (107.03 to 122.91)	120.73 (111.69 to 129.77)	**0.03**	0.66
More than 30 years of experience	*N* = 5	117 (105.53 to 128.47)	121.6 (115.74 to 127.46)	0.49	0.51

^a^ Paired-samples t-test

The bold numbers are significant *P*-values (*p* < 0.05)

### Comparison of Oral health assessment tool (OHAT) scores

Among the 132 patients, the post-training response rate was 73% (96 patients). The characteristics of the 132 patients are described in Table 2. In the second OHAT survey, participants were excluded due to the following: non-response (36 patients), missing OHAT data (13 patients), hospital admission (2 patients), death (2 patients), and no diagnosis of dementia (8 patients). Seventy-one patients were assessed after these adjustments. Among the 71 patients whose post-training were included, 53 (74.6%) patients were women, 27 (38.0%) were 75–84 years old, and 40 (56.3%) were over 85 years old. The most common cause of dementia was Alzheimer’s disease (*n* = 41, 57.7%). The results of the comparison of the pre-training and post-training OHAT scores of the 71 patients are shown in Table 3. There were significant improvements in the tongue (from 0.80 to 0.59, *P* < 0.05), natural teeth (from 1.41 to 1.32, *P* < 0.05), and oral hygiene (from 1.44 to 1.15, *P* < 0.05) scores.

**Table 2:** Patient characteristics

	Total (*n* = 132)	Data available (*n* = 71)	Lost to follow up^a^ (*n* = 61)	*P*-value^b^
Age, *n* (%)				
< 65 years	1 (0.8)	0 (0.0)	1 (1.6)	0.46
65–74 years	11 (8.3)	4 (6.0)	7 (11.5)	0.56
75–84 years	51 (38.6)	27 (38.0)	24 (39.3)	0.67
≥ 85 years	68 (51.5)	40 (56.3)	28 (45.9)	0.39
Women, *n* (%)	96 (72.7)	53 (74.6)	43 (70.5)	0.70
Aetiology of dementia, *n* (%)				
Alzheimer’s disease	55 (41.7)	41 (57.7)	14 (23.0)	< 0.001
Lewy body dementia	11 (8.3)	7 (9.9)	4 (6.6)	0.54
Vascular dementia	8 (6.1)	5 (7.0)	3 (2.3)	0.72
Other type of dementia	7 (5.3)	4 (5.6)	3 (2.3)	1.00
Dementia with undetermined aetiology	43 (32.6)	14 (19.7)	29 (47.5)	< 0.001

^a^ Lost to follow up: 36 patients were excluded due to non-response; 12 patients were excluded due to missing OHAT data; 1 patient was excluded due to missing data on age; 2 patients were excluded due to hospital admission; 2 patients were excluded due to death; and 8 patients were excluded due to no diagnosis of dementia

^b^ The Mann-Whitney U test and Fisher’s exact test were used to compare the available data and the data of those lost to follow up. The threshold for statistical significance was set at *p* < 0.05

**Table 3:** Mean pre-training and post-training Oral Health Assessment Tool scores (*n* = 71)

Category	Pre-training (*n* = 71)	Post-training (*n* = 71)	*P*-value
Lips	0.55	0.54	0.88^a^
Tongue	0.80	0.59	**0.04**^**a**^
Gums and tissues	0.86	0.80	0.64^a^
Saliva	0.66	0.59	0.48^a^
Natural teeth	1.41 (*n* = 63)	1.32 (*n* = 63)	**0.02**^**a**^
Dentures	0.81 (*n* = 31)	0.80 (*n* = 30)	0.94^a^
Oral hygiene	1.44	1.15	**0.01**^**a**^
Dental pain	0.70	0.59	0.36^a^

^a^ Wilcoxon signed rank test

The bold numbers are significant *P*-values (*p* < 0.05)

## Discussion

The findings of this study show that the multimodal comprehensive care methodology training for oral health care professionals improved their empathy for patients with dementia.

This study is the first to investigate the development of empathy for patients with dementia among oral health care professionals. Previous studies demonstrated that web-based interventions improved informal caregivers’ empathy for patients with dementia [13, 14]. However, informal caregivers are generally not involved in dental care and treatment. Other studies demonstrated that completing a person-centred course and adding patient videos to a lecture improved dental students’ empathy [22, 23]. Although many clinical studies have been conducted on empathy interventions, the majority of such interventions have targeted medical students and residents [24]. Therefore, our intervention aimed to improve the empathy of oral health care professionals. Considering the importance of the clinical impact on reducing the care-resistant behaviour of patients with dementia and improving communication skills in the relationship between oral health care professionals and patients during oral care or treatment, further studies are needed to improve oral health care professionals’ empathy for patients with dementia.

Our study showed that regardless of the gender and years of clinical experience of the participants, all post-training subgroup scores were higher than the pre-training subgroup scores. However, there was no statistically significant difference in some subgroups, i.e., male participants and participants with less than 11 years of experience or more than 30 years of experience. The present results suggest that multimodal comprehensive care methodology training may improve empathy regardless of the number of years of clinical experience. Further studies with larger samples are needed to clarify this association.

We believe that there are two reasons why the multimodal comprehensive care methodology training improved the empathy of oral health care professionals. First, this methodology emphasizes both verbal and non-verbal communication skills for both caregivers and care receivers. Past studies have shown that interventions based on the neurobiology of oral care-resistant behaviour also target both verbal and non-verbal communication skills [6, 7]. Second, this methodology training focuses on not only lectures about dementia-related knowledge and care but also specific practical care strategies based on a humane philosophy and neurological theory of dementia. Interventions that focus more on enhancing dementia-related knowledge and care through lectures might lead to more negative attitudes towards people with dementia [13]. It has been proposed that interventions related to people with dementia should be designed so that caregivers feel a sense of competence and perceive that their knowledge and skills related to caring for people with dementia are growing rather than focusing on increasing dementia-related knowledge [25].

Our study showed post-training improvement in the oral health of patients with dementia who had poor oral health due to the refusal of usual oral care or dental treatment. The improvements in the tongue and oral hygiene scores may suggest that the improvements in the participants’ empathy decreased the oral health care resistance behaviour of the identified patients who had previously refused usual oral health care or dental treatment. In addition, natural teeth scores are improved by conservative treatment of dental caries by dentists’ professional intervention. The improvements in natural teeth scores observed in this study may have occurred because dental treatments, such as the treatment of dental caries, were performed post-training, as there were more dentists than dental hygienists in this study. The improved empathy of the participating dental hygienists may also have enabled treatment by dentists. However, our results regarding the OHAT scores are of serious concern given that 61 patients were lost to follow up among the 132 patients who resisted usual oral care or dental treatment whom the participants listed. Before the participants in this study were recruited, it was assumed that patients who refused usual oral care or dental treatment might not have been able to receive oral care or dental treatment after 1 month because they could have advanced dementia. In terms of patient characteristics, more of the patients whose post-training data were included had Alzheimer’s disease than the patients who were lost to follow up. On the other hand, there were no significant age or gender differences between those whose post-training data were included and those who were lost to follow up. Regarding the patient characteristics, the OHAT results assessed as post-training data were likely to lead to the overestimation of the intervention effect. Further studies are needed with a control group to determine the relevance of the intervention.

Several limitations should be discussed. First, this study was a pre-post prospective study, not a randomized controlled trial. Therefore, it is possible that confounding factors influenced the association between the training and the JSPE-HP and OHAT scores. Second, the sample size of this study was small. Third, measurements of empathy in a medical population may be subject to significant social desirability bias; therefore, particularly with self-report measures of empathy, it can be difficult to assert whether training or awareness of the desirability of an empathetic physician increases empathy [24]. Self-report surveys can be an effective and reliable measure of physician empathy, but they must be validated against behavioural or patient-report measures. Fourth, the internal consistency reliability of the JSPE scores was low, which could be related to the small sample size; future studies with large numbers of participants are needed. Fifth, the outcome assessment time frame in this study might be a weakness with respect to assessing empathy. While health professionals’ empathy may significantly increase immediately after training, there are limited insights about the long-term efficacy of the training [24]. Sixth, the OHAT scores were calculated by the same oral health professionals in both the pre-training and post-training assessments. These results could have been influenced by a placebo effect.

## Conclusions

This study is the first to assess the effectiveness of multimodal comprehensive care methodology training in improving the empathy of oral health care professionals. The multimodal comprehensive care methodology training was associated with an improvement in oral health care professionals’ empathy for patients with dementia. Because this was a single arm pre-post study, the findings suggest that randomized controlled trials with large sample sizes will be needed.

## Data Availability

The datasets used during the current study are available from the Tokyo Metropolitan Geriatric Hospital, but restrictions apply regarding the availability of these data, and they are not publicly available. However, the data are available from the corresponding author upon reasonable request and with permission of Tokyo Metropolitan Geriatric Hospital.

## Abbreviations

JSPE-HP: Jefferson Scale of Physician Empathy-Health Professionals Version
OHAT: Oral Health Assessment Tool
